# Retrospective assessment of self-reported exposure to medical ionizing radiation: results of a feasibility study conducted in Germany

**DOI:** 10.1186/s13104-015-1268-8

**Published:** 2015-07-10

**Authors:** Steffen Dreger, Saskia Poettgen, Florence Samkange-Zeeb, Hiltrud Merzenich, Anye Ningo, Joachim Breckow, Hajo Zeeb

**Affiliations:** Leibniz Institute for Prevention Research and Epidemiology-BIPS, Achterstrasse 30, 28359 Bremen, Germany; Institute of Medical Biostatistics, Epidemiology and Informatics, University Medical Centre Mainz, Obere Zahlbacher Strasse 69, 55131 Mainz, Germany; Technische Hochschule Mittelhessen (THM), University of Applied Sciences, Wiesenstrasse 14, 35390 Giessen, Germany; Health Sciences Bremen, University of Bremen, Bibliothekstrasse 1, 28359 Bremen, Germany

**Keywords:** Medical radiation exposure, German national cohort, Feasibility study, Assessment of self-reporting, Health insurance data

## Abstract

**Background:**

Exposure to medical ionizing radiation has been increasing over the past decades and constitutes the largest contributor to overall radiation exposure in the general population. While occupational exposures are generally monitored by national radiation protection agencies, individual data on medical radiation exposure for the general public are not regularly collected. The aim of this study was to determine the feasibility of assessing lifetime medical ionizing radiation exposure from diagnostic and therapeutic procedures retrospectively and prospectively within the framework of the German National Cohort study.

**Methods:**

Retrospective assessment of individual medical radiation exposure was done using an interviewer-based questionnaire among 199 participants (87 men and 112 women) aged 20–69 randomly drawn from the general population at two recruitment locations in Germany. X-ray cards were distributed to 97 participants at one recruitment center to prospectively collect medical radiation exposure over a 6-month period. The Wilcoxon–Mann–Whitney test was used to test differences in self-reported median examination frequencies for the variables age, sex, and recruitment center. To evaluate the self-reported information on radiological procedures, agreement was assessed using health insurance data as gold standard for the time period 2005 to 2010 from 8 participants.

**Results:**

Participants reported a median of 7 lifetime X-ray examinations (interquartile range 4–13), and 42% (n = 83) reported having had a CT scan (2, IQR = 1–3). Women reported statistically significant more X-ray examinations than men. Individual frequencies above the 75th percentile (≥15 X-ray examinations) were predominantly observed among women and in individuals >50 years of age. The prospective exposure assessment yielded a 60% return-rate of X-ray cards (n = 58). 16 (28%) of the returned cards reported radiological examinations conducted during the 6-month period but generally lacked more detailed exposure information. X-ray examinations reported for the period for which health insurance data were available provided a moderately valid measure of individual medical radiation exposure.

**Conclusions:**

The assessment of more recent medical examinations seems in the German National Cohort study feasible, whereas lifetime medical radiation exposure appears difficult to assess via self-reports. Health insurance data may be a potentially useful tool for the assessment of individual data on medical radiation exposure both retrospectively and prospectively.

**Electronic supplementary material:**

The online version of this article (doi:10.1186/s13104-015-1268-8) contains supplementary material, which is available to authorized users.

## Background

With the advancement of medical science and health care technologies, diagnostic imaging techniques and interventional radiological procedures are increasingly used to accurately diagnose a wide range of diseases and injuries. In particular, the frequency of dose-intensive diagnostic procedures such as computed tomography (CT) has been increasing worldwide, for instance in the United States [1] and in Germany [2]. In Germany, the annual radiation exposure from medical diagnostic examinations gradually increased from 1.5 to 1.8 mSv per capita dose from 1996 to 2010. Much larger increases have been noted in the US [3]. In both countries, this is mainly due to the concurrent increase of CT examinations. While CT examinations account for only 6% of all types of medical examinations, they contribute approximately 60% of the collective effective dose of medical diagnostic exposures in Germany [4].

Collecting individual medical radiation exposure data for the general public in many countries including Germany is a complex endeavor, as there is no regular patient monitoring and surveillance of ionizing radiation exposure. In recent international studies, diagnostic and therapeutic examination data for individuals were abstracted from hospital radiological databases to assess and analyze individual radiation exposure and health outcomes such as cancer or cardiovascular diseases [5, 6]. Most hospital databases however fail to provide a comprehensive overview of lifetime medical radiation exposure. Additionally, there are difficulties in estimating doses due to limited dose information. Thus most studies primarily rely on published dose estimates for exposure assessment, e.g. [7].

When collecting medical examinations using self-reported information, assessing the reliability of the collected data with regard to actual received procedures and associated radiation exposure is essential. In previous international studies, medical records were used to assess the agreement of self-reports for radiological examinations and radiation exposures [8–11]. In non-clinical, population-based studies, health insurance data may provide similar validation options for self-reporting where such data are available, and may additionally provide data of all received radiological examinations covered by the health insurance provider [12]. Validation with medical records or health insurance data as the gold standard may identify the potential introduction of recall or other bias which may in turn lead to under-/over-reporting in the self-reported data. For such purposes, comprehensive health insurance data have been available for health research in Germany since 2005, and comprise out-patient and hospital service records including procedures using medical ionizing radiation.

The main aim of this project was to develop and test tools to assess radiation exposures from diagnostic and therapeutic procedures retrospectively and prospectively for a comprehensive assessment of individual lifetime medical radiation exposure. To identify potential discrepancies in self-reporting, agreement between health insurance data and self-reports were also calculated. This study was part of several feasibility studies conducted in preparation for the German national cohort study. The prospective, long-term population-based cohort study aims to recruit 200,000 participants aged 20–69 years. The participants will be medically examined and questioned about their living habits with the aim of investigating causes, risk factors, preventive potentials and early detection approaches for a wide range of chronic and infectious diseases [13].

## Methods

### Retrospective and prospective medical radiation exposure assessment

A questionnaire to retrospectively assess previous ionizing medical radiation exposure was developed based on survey instruments used in previous radiation-epidemiological studies addressing exposure to medical diagnostic and therapeutic radiation [14–16]. The questionnaire aimed to assess individual lifetime exposure to medical radiation from conventional X-ray examinations, as well as intensive examinations such as computed tomographies (CT), interventional radiological examinations e.g. heart catheterization, nuclear medicine examinations including scintigraphy, and radiation therapy (Additional file 1).

X-ray cards were designed for prospective assessment. In the event of a radiological examination, the attending physician was asked to provide exposure-related data (i.e. type of examination, body part, if possible: dosimetry information such as dose length product (DLP)) on the card. For logistic reasons the X-ray cards were distributed only at one recruitment center (Bremen) to use for a period of 6 months.

Participants for this feasibility study were 20–69 years old. The sample was randomly drawn from the residents’ registration offices at two recruitment centers in Bremen and Hamburg, Germany, stratified by age and sex. The questionnaire survey was conducted between 2011 and 2012, during the feasibility phase of the German national cohort study. The interviews were administered by specially trained study nurses and were conducted in recruitment centers specifically set up for the purpose of this study, not in hospitals.

Survey results were stratified by age, sex, and type of examination. We only report numbers for conventional X-ray and computed tomography procedures because they account for most frequent diagnostic examinations. Dental radiological examinations are reported but excluded for the statistical analyses as these examinations contribute minimal radiation doses compared to other examinations such as CT scans. We additionally conducted sub-analyses of individuals who were in the 75th percentile of reported examinations in the study population (i.e., had more than 15 examinations in their lifetime), whom we classified as highly exposed. The Wilcoxon–Mann–Whitney test was used to test differences in self-reported median examination frequencies for the variables age, sex, and recruitment center. Data management and statistical analyses were done using SAS 9.3 (SAS Institute, Cary, NC, USA).

### Assessment of self-reporting reliability

97 of 99 of the participants in Bremen consented to the abstraction of their health insurance data for the validation of their reported information. However, only one insurance company agreed to collaborate and provided claims data for 2004–2010. To assess the agreement between self-reporting and health insurance data as the gold-standard, we calculated a simple overall agreement measure, dividing the actual examinations recorded in the health insurance data by the self-reported examinations.

## Results

### Medical radiation exposure assessment

In total, 199 individuals (Bremen n = 99, Hamburg n = 100) participated in this study, 87 male and 112 female (Table 1). The sex distribution was similar in both study centers. The mean age of all participants was 48.5 years (range 20–69) and the mean interview duration was 11 min (standard deviation ±5 min).

**Table 1:** Study population characteristics

	Total study population (n = 199)		Highly exposed individuals (≥15 examinations) (n = 51)	
	n	%	n	%
Sex				
Female	112	56.3	33	64.7
Male	87	43.7	18	35.3
Age (in years)				
<40	59	29.6	6	11.8
40–50	40	20.1	7	13.7
>50	100	50.3	38	74.5
Center				
Bremen	99	49.7	27	52.9
Hamburg	100	50.3	24	47.1

### Conventional X-ray examinations

All participants reported having had at least one radiological examination. Most participants reported having had examinations of the upper (n = 122) and lower extremities (n = 118), thorax region (n = 112), spine (n = 87), and mammograms in women (n = 60) (Table 2). The median number of examinations was 7 (interquartile range 4–13); women reported statistically significant more X-ray examinations than men: 8 (IQR 4–14) and 6 (IQR 3–11), respectively (Table 2). The number of reported X-ray examinations increased with age: the above 50 age group reported significantly more X-ray examinations with a median of 10 (IQR 5–15) compared to the under 40 [4 (IQR 2–7)] and 40–50 years age groups [7 (IQR 4–10)], respectively (Table 2). 60 female participants (54%) reported having had a mammogram (Table 2); about 25% of these women were below the age of 50. The median number of reported mammograms was 3 (IQR 2–6) and increased with age.

**Table 2:** Median number of self-reported examinations for X-ray and computed tomography examinations by total study population and highly exposed participants

	Total study population (n = 199)				Highly exposed individuals (>=15 examinations) (n = 51)			
	X-ray examinations (N = 199)		CT examinations (N = 83)		X-ray examinations (N = 51)		CT examinations (N = 51)	
	n	Median (IQR)	n	Median (IQR)	n	Median (IQR)	n	Median (IQR)
Body part		7.0 (4.0–13.0)		2.0 (1.0–3.0)		17.0 (14.0–27.0)		2.0 (2.0–5.5)
Head	81	2.0 (1.0–3.0)	40	1.0 (1.0–1.0)	28	2.0 (1.0–5.0)	14	1.0 (1.0–2.0)
Thorax	112	2.0 (1.0–4.5)	15	1.0 (1.0–6.0)	41	5.0 (2.0–8.0)	10	4.5 (1.0–7.0)
Abdomen	31	1.0 (1.0–1.0)	15	1.0 (1.0–5.0)	16	1.0 (1.0–2.5)	11	1.0 (1.0–7.0)
Spine	87	2.0 (1.0–3.0)	27	1.0 (1.0–2.0)	41	2.0 (1.0–4.0)	14	1.5 (1.0–2.0)
Lower extremities	118	2.0 (1.0–3.0)	12	1.5 (1.0–2.0)	41	3.0 (1.0–4.0)	5	1.0 (1.0–2.0)
Upper extremities	122	2.0 (1.0–3.0)	2	1.5 (1.0–2.0)	36	2.0 (2.0–4.0)	2	1.5 (1.0–2.0)
Mammograms (females only)	60	3.0 (2.0–6.0)	–	–	28	5.5 (2.5–8.5)	–	–
Sex								
Female	112	8.0 (4.0–14.0)*	35	2.0 (1.0–4.0)	33	17.0 (15.0–24.0)	20	2.5 (2.0–5.5)
Male	87	6.0 (3.0–11.0)*	48	2.0 (1.0–3.0)	18	16.5 (14.0–20.0)	16	2.0 (2.0–7.0)
Age (in years)								
<40	59	4.0 (2.0–7.0)*	14	1.0 (1.0–2.0)	6	21.0 (18.0–26.0)*	4	2.5 (1.5–5.0)
40–50	40	7.0 (4.0–10.0)*	17	2.0 (1.0–2.0)	7	16.0 (14.0–30.0)*	5	2.0 (2.0–4.0)
>50	100	10.0 (5.0–15.0)*	52	2.0 (1.0–3.5)	38	17.0 (14.0–24.0)*	27	2.0 (2.0–6.0)
Center								
Bremen	99	7.0 (4.0–13.0)	39	1.0 (1.0–3.0)	27	17.0 (14.0–23.0)	18	2.0 (1.0–3.0)*
Hamburg	100	7.0 (4.0–13.0)	44	2.0 (1.0–2.0)	24	17.0 (14.0–24.5)	18	3.5 (2.0–6.0)*

*IQR* interquartile range.

*Significant at p value <0.05.

### High dose examinations

83 study participants (42%) reported ever having had a CT examination [median number of exams 2.0 (IQR 2.0–5.5)] (Table 2). The most participants reported having had CT examinations of the head (n = 40) followed by spine (n = 27), abdomen (n = 15) and thorax (n = 15) examinations (Table 2). Interventional examinations (e.g. cardiac catherization or stent insertion), nuclear medicine examinations and radiation therapy treatment were infrequent (data not shown).

### Highly exposed individuals

51 (25.6%) of the participants had examinations in the 75th percentile (more than or equal to 15 lifetime X-ray examinations) and were therefore classified as highly exposed. In this group, 33 (64.7%) were female and 38 (74.5%) more than 50 years of age, and were equally distributed between both centers (Table 1). This group accounted for 38% of all reported X-ray examinations in the overall study population. With regard to computed tomography scans these individuals accounted for almost half of all reported CT examinations in the overall study population [73% for abdominal CT, 52% for spine CT (Table 2)]. The median number of examinations for X-ray and computed tomography examinations among this group were 17 (IQR 14–27) and 2 (IQR 2.0–5.5), respectively (Table 2). Females reported more CT examinations than males (2.5, IQR 2.0–5.5 vs. 2.0, IQR 2.0–7.0; p = 0.35), and participants more than 50 of age reported more CT examinations than younger participants (p = 0.48) (Table 2). Participants in Hamburg (66.7% of whom were female) reported significantly more CT examinations compared to those in Bremen: median 3.5 (IQR 2–6) and 2.0 (IQR 1–3), respectively.

### Prospective exposure assessment

X-ray cards for the prospective assessment were distributed to 98 Bremen participants. One participant did not accept the X-ray card. A total of 58 participants returned the cards to the Bremen recruitment center after the 6-month period as required. 16 (28%) of the returned cards provided basic data such as body part examined, but generally lacked more detailed exposure information such as dose area/-length product values required for detailed (organ) dose-estimations. In total, 9 women and 7 men reported 23 radiological examinations during the 6 months of the study. The mean age of the respondents was 56.3 years (range 32–68). The most frequently reported examination was dental X-ray (n = 10), the spine (n = 3), and intravenous urograms (n = 3). Additionally, one mammogram and two head CT examinations were reported (data not shown.)

### Agreement between health insurance data and self-reported information

One insurance company with eight participating insurants (all female, mean age 56.1; 32–68) agreed to collaborate and provide data on medical ionizing radiation examinations for the period 2005–2010.

The participants reported a total of 41 X-ray examinations for this period, most of them conventional X-rays. The single most reported examination was mammograms. Two individuals reported having had a computed tomography examination. According to the health insurance data, the participants had undergone a total of 38 examinations, of which the participants actively reported 28 examinations only (74% of 38). Of the ten examinations found in the health insurance data but not reported by the participants, seven (70%) were conventional X-ray examinations and three (30%) were computed tomographies. Among the 13 self-reported examinations that were not found in the health insurance data, the most frequent examinations were mammograms (5; 38%) and computed tomographies (3; 23%) (data not shown).

Overall, a moderate agreement of 55% was found between self-reporting and health insurance data for the time period 2005–2010. The proportion of agreement decreased with increasing time since examination. Reported examinations conducted during the 24 months prior to the survey had a 67% agreement rate with insurance data. Rates remained stable at approximately 65% until 2008 and then dropped to below 60% agreement for examinations reported between 2007 and 2005.

## Discussion

We assessed individual medical radiation exposure using a retrospective interviewer-based questionnaire approach among 199 participants aged 20–69 at two recruitment sites in Germany. All participants reported having had at least one X-ray examination, and 42% reported having had a CT examination during their lifetime. The majority of participants classified as highly exposed were female and older than 50 years of age. Our explorative assessment of self-reporting reliability indicated that self-report of radiological examinations during the 6-year period 2005–2010 generally provided a moderately accurate account of individual medical radiation exposure. The agreement however decreased over time.

A strength of the standardized personal interview approach is that it allowed us to probe participants and also to offer additional explanations and descriptions of the different examinations [e.g. the difference between CT and magnetic resonance imaging (MRI)] as required. This is a major advantage in comparison to self-administered questionnaires [14–16]. Furthermore, we used health insurance data to validate self-reporting of radiological examinations for a subsample of the study participants. In contrast to medical data such as hospital records, insurance health data in Germany include every radiological examination covered by the respective insurance provider during the time period under study, providing a comprehensive and valid account of X-ray examinations. We were thus able to assess accuracy of year and type of examination reported by the subsample of participants.

As a limitation, we were restricted to a small subsample (n = 8) from one German insurance provider for the self-reporting assessment, as the other insurance companies did not consent to our request for data. Furthermore, we could only assess the period 2005–2010 because health insurance data were not available for the years earlier than 2005. The subsample included only women and the number of regular (bi-) annual mammogram examinations (which are covered by insurance via the German breast cancer screening program for the age group 50–69) may have had an effect on the moderate correlation between health insurance data and self-reported examinations. Due to the limited data for assessing the agreement between health insurance data and self-reporting we could only use a simple correlation measure. However, we additionally calculated the intra-class correlation coefficient, which presented similar results. Access to health insurance data requires an extensive formal approval process due to very strict data protection regulations in Germany. For this feasibility study, we started the formal approval process once data collection was completed and concrete numbers on health insurance coverage were available to contact the respective providers; hence, the ad hoc approval requests were difficult and resulted in the health insurance providers’ very low willingness to cooperate. For the main German National Cohort study, however, these permissions are expected to be in place. Health insurance data and additional secondary data will be available for participants who consent to data abstraction.

The medical radiation examination frequencies observed in this survey are consistent with data from the German Federal Office for Radiation Protection indicating that high-dose examinations such as computed tomographies are increasingly contributing to the total per capita dose in Germany [4]. It should however be noted that because of the age inclusion criteria of the German National cohort (20–69 years of age), the observed exposure patterns are only applicable to the adult population in Germany.

The results of our sub-analysis to assess self-reporting reliability in our study should be carefully interpreted due to the sparse data provided by one insurance company only. The differences we saw in self-reporting and insurance data resulted mainly from under-reporting, which has been also observed in earlier international studies and related to underreporting of high-dose examinations such as computed tomographies, and accurate reporting of routine procedures such as mammograms [8–11]. Some age effects in our study may have additionally affected the self-reporting as we observed some under-reporting from relatively old participants (mean 56.1 years) [9]. In addition to the observed under-reporting as indicated by the health insurance data, we additionally found some evidence of over-reporting in our data. These examinations were reported by the participants but were not recorded in the health insurance data. Similar to under-reporting, this also involved mammograms and CT examinations. As CT examinations are rather expensive and in Germany it is generally not common to pay for such medical examinations privately, we therefore surmise that these extra CT examinations are factual over-reporting, possibly being mixed up with MRI. We have no explanation for the additional, over-reported mammograms in our study sample. In practice in Germany, mammograms outside the screening program are mostly conducted based on specific indications such as family history of breast cancer or palpable lump. These examinations are fully covered by the insurance provider and should therefore appear in the health insurance data.

Estimations of organ or effective doses from the examinations which were done remain another major issue. A subproject of this feasibility study was a dosimetry study to explore whether it is possible to do dose estimations based on the information available. The questionnaires and X-ray cards provided only basic information on the part of body examined as well as frequency and date of examination. Similarly, the insurance data provided only limited relevant information. We thus arrived at rather crude dose estimates, which are not reported here. Linkage with other data sources, e.g. hospital databases, could provide further options to arrive at comprehensive assessment of individual medical radiation exposure. For example, Chen and colleagues in Canada suggest the implementation of so-called patient exposure registers [17]. Additionally, the International Atomic Energy Agency proposes a smart card approach to prospectively monitor radiation exposure and collect detailed dosimetry information for more precise dose estimates [18, 19].

Future research could incorporate web-based questionnaires with multimedia input options such as images, animations or short video clips to facilitate better understanding of technical terminology and precise reporting of specific examinations for subsequent radiation exposure estimates. Web-based approaches are likely to be less resource-intensive in comparison to interviewer-based surveys, and could even be implemented population-wide nationally and, perhaps, internationally. Population-wide, web-based approaches may, however, not be equally accessible by all population groups; hence selection bias remains a potential limitation. Complementary to the prospective exposure assessment using X-ray cards, quarterly web-based questionnaires could be implemented as an inexpensive tool to collect individual information on radiological examinations during the follow-up period. To address the observed over-reporting issue, additional questions regarding out-of pocket payments should be included in future questionnaires to account for potential examinations not covered by the health insurance company. To control for potential reporting bias factors affecting the reporting such as educational level and socio-economic status should be considered in further analyses as part of the main study. Furthermore, insurance providers should be involved at a very early stage in future projects to ensure cooperation and sufficient data for more reliable assessment of self-reporting.

In summary, this study indicates that while individuals can relatively accurately recall previous X-ray examinations for the period of up to 4 years, it is difficult to assess lifetime medical radiation exposure using self-report. It further shows that—provided legal and data protection requirements are met—health insurance data are a viable and potentially valid source of information for the study and for monitoring of medical ionizing radiation exposure in the general population. This is especially so as it is expected that data from several health insurance companies will be available in the main German National Cohort. Furthermore, the possibility of linkage with hospital databases for more precise dose information is also expected to increase.

## Additional file

Additional file 1: Retrospective questionnaire to assess medical radiation exposure within the German National Cohort.
