# Supplementary material for: Retrospective assessment of self-reported exposure to medical ionizing radiation: results of a feasibility study conducted in Germany
Source: BMC Res Notes. 2015 Jul 10;8:300. doi: 10.1186/s13104-015-1268-8 (PMC4702423; doi:10.1186/s13104-015-1268-8)
Supplement: Additional file 1: — Retrospective questionnaire to assess medical radiation exposure within the German National Cohort. [file 13104_2015_1268_MOESM1_ESM.pdf]

Universität Bremen

BIPS

ID Number: |__|__|__|__|__|__|__|

Feasibility study to assess medical radiation exposure within the German National Cohort

U-ID: |__|__|__|__|

Date of interview: _____/______/2011

Date of interview „Medical Radiation Exposure“: ____**:**____

I would like to ask you a few questions regarding different diagnostic or therapeutic x-rays examinations which you may have had in the past. At times I will use medical terms, but will always explain these to you.

Should any of these terms however remain unclear to you, please let me know.

We are interested in all medical x-ray examinations or other radiation-related applications which you may have had or undergone in the past.

Computed Tomography (CT)

I would like to begin with questions on computed tomography (CT) examinations.

During CT examinations the patient lies on a table which slowly glides into a ring-shaped imaging device. This procedure generally takes a few minutes. There are no loud knocking sounds to be heard as is the case with magnetic resonance imaging (MRI) procedures. You are not asked if you have any metallic parts in your body, such as pacemakers. Furthermore, you are not asked to take off any metal items such as wristwatches.

(➔*If necessary, show image*)

A0 Did you <u>ever</u> have a computed tomography (CT) examination?
Please also think of any past events such as severe traffic accidents, head injuries, spine injuries, broken bones, slipped disc, joint inflammation, respectively arthritis, or spine deformities such as scoliosis. Please also think about infectious illnesses such as pneumonia/infection of the lungs or appendicitis, and severe diseases such as cancer. CT scans are conducted in preparation for surgical operations.

☐ 1 **Yes** ⇨ continue with A1.0

☐ 2 **No** ⇨ continue with B0

☐ -8 **Don’t know** ⇨ continue with B0

Computed Tomography

A1.0 Please try to remember <u>all</u> CT examinations you ever had.

Which examinations were conducted? I will read out different examinations. Please respond to each possibility.

Info for interviewer: Please go through the list of body parts, even if the subject names a specific examination right away.

For each examination the total number will be asked first, followed by the respective year, beginning with the most recent (e.g. 2013).

**If the subject cannot remember the year use the probing question: How old were you (in years)?*

For paper version: Where there are more than 3 examination years per examination, please enter the other years in the area "further examinations" at the end of the section.

Did you have a CT scan of the…	Yes	No	Don't know	How often in total	In which year, beginning with the most recent one?	At what age?	How often?
Head, Skull	☐1	☐2	☐-8	|__|__|	|__|__|__|__| |__|__|__|__| |__|__|__|__|	|__|__| |__|__| |__|__|	|__|__<br>|__|__<br>|__|__
Soft tissues of the neck	☐1	☐2	☐-8	|__|__|	|__|__|__|__| |__|__|__|__| |__|__|__|__|	|__|__| |__|__| |__|__|	|__|__<br>|__|__<br>|__|__
**Thoracic region**<br>(Lungs, Heart)	☐1	☐2	☐-8	|__|__|	|__|__|__|__| |__|__|__|__| |__|__|__|__|	|__|__| |__|__| |__|__|	|__|__<br>|__|__<br>|__|__
Abdominal region	☐1	☐2	☐-8	|__|__|	|__|__|__|__| |__|__|__|__| |__|__|__|__|	|__|__| |__|__| |__|__|	|__|__<br>|__|__<br>|__|__
Angiography	☐1	☐2	☐-8	|__|__|	|__|__|__|__| |__|__|__|__| |__|__|__|__|	|__|__| |__|__| |__|__|	|__|__<br>|__|__<br>|__|__
Shoulder/Pelvis/<br>Neck of femur	☐1	☐2	☐-8	|__|__|	|__|__|__|__| |__|__|__|__| |__|__|__|__|	|__|__| |__|__| |__|__|	|__|__<br>|__|__<br>|__|__

Did you have a CT scan of the…	Yes	No	Don't know	How often in total	In which year, beginning with the most recent one?	At what age?	How often?
Upper extremities such as forearm, wrist	☐1	☐2	☐-8	|__|__|	|__|__|__|__| |__|__|__|__| |__|__|__|__|	|__|__| |__|__| |__|__|	|__|__| |__|__| |__|__|
Lower extremities such as femur/ knee/shinbone	☐1	☐2	☐-8	|__|__|	|__|__|__|__| |__|__|__|__| |__|__|__|__|	|__|__| |__|__| |__|__|	|__|__| |__|__| |__|__|
Spine, whole *(if necessary, please ask whether the whole spine (i.e. cervical/thorax and lumbar spine) was examined. If not, please fill out each single spinal region.*	☐1	☐2	☐-8	|__|__|	|__|__|__|__| |__|__|__|__| |__|__|__|__|	|__|__| |__|__| |__|__|	|__|__| |__|__| |__|__|
Lumbar spine	☐1	☐2	☐-8	|__|__|	|__|__|__|__| |__|__|__|__| |__|__|__|__|	|__|__| |__|__| |__|__|	|__|__| |__|__| |__|__|
Cervical spine	☐1	☐2	☐-8	|__|__|	|__|__|__|__| |__|__|__|__| |__|__|__|__|	|__|__| |__|__| |__|__|	|__|__| |__|__| |__|__|
Thoracic spine	☐1	☐2	☐-8	|__|__|	|__|__|__|__| |__|__|__|__| |__|__|__|__|	|__|__| |__|__| |__|__|	|__|__| |__|__| |__|__|
**Other body parts**<br>_______________	☐1	☐2	☐-8	|__|__|	|__|__|__|__| |__|__|__|__| |__|__|__|__|	|__|__| |__|__| |__|__|	|__|__| |__|__| |__|__|

Further examinations:

Body part	In which year?	At what age?	How often?

Space for interviewer's comments:

Computed tomographies

A2.0 Did you <u>ever</u> have a so-called PET-CT (Positron-Emission-Tomography-CT)?

This is a computed tomography examination during which a radioactive agent is injected into the body, e.g. so that a tumor is visible.

☐1 **Yes** ⇨ continue with A*2.1*

☐2 **No** ⇨ continue with *B0*

☐-8 **Don't know** ⇨ continue with *B0*

Info for interviewer: Please go through the list of body parts, even if the subject names a specific examination right away.

For each examination the total number will be asked first, followed by the respective year, beginning with the most recent (e.g. 2013).

**If the subject cannot remember the year use the probing question: How old were you (in years)?*

For paper version: Where there are more than 3 examination years per examination, please enter the other years in the area "further examinations" at the end of the section.

A2.1

How often in total?	In which year, beginning with the most recent one?	At what age?	How often?
|__|__|	|__|__|__|__|	|__|__|	|__|__|
	|__|__|__|__|	|__|__|	|__|__|
	|__|__|__|__|	|__|__|	|__|__|

Further PET- CT:

In which year?	At what age, respectively?	How often?

Space for interviewer's comments:

X-ray examinations

B0 We now come to common X-ray examinations.

Did you ever have an x-ray examination?

Please also consider past screening x-ray examinations, e.g. for tuberculosis.

Perhaps you remember breaking a bone, having a chest infection, being hospitalised or having an operated.

☐ 1 **Yes** ⇨ continue with B1.0

☐ 2 **No** ⇨ continue with B2.0

☐ -8 **Don't know** ⇨ continue with B2.0

X-ray examinations

B1.0 What kinds of examinations were conducted? I read to you different examination possibilities. Please answer to each item.

Info for interviewer: Please go through the list of body parts, even if the subject names a specific examination right away.

For each examination the total number will be asked first, followed by the respective year, beginning with the most recent (e.g. 2013).

**If the subject cannot remember the year use the probing question: How old were you (in years)?*

For paper version: Where there are more than 3 examination years per examination, please enter the other years in the area "further examinations" at the end of the section.

Did you have an x-ray examination of the following body parts …	Yes	No	Don't know	How often in total	In which year, beginning with the most recent one?	At what age?	How often?
Skull	☐1	☐2	☐-8	|__|__|	|__|__|__|__| |__|__|__|__| |__|__|__|__|	|__|__| |__|__| |__|__|	|__|__| |__|__| |__|__|
Jawbone	☐1	☐2	☐-8	|__|__|	|__|__|__|__| |__|__|__|__| |__|__|__|__|	|__|__| |__|__| |__|__|	|__|__| |__|__| |__|__|
Paranasal sinuses	☐1	☐2	☐-8	|__|__|	|__|__|__|__| |__|__|__|__| |__|__|__|__|	|__|__| |__|__| |__|__|	|__|__| |__|__| |__|__|
Nose	☐1	☐2	☐-8	|__|__|	|__|__|__|__| |__|__|__|__| |__|__|__|__|	|__|__| |__|__| |__|__|	|__|__| |__|__| |__|__|
Orbit (Eye socket)	☐1	☐2	☐-8	|__|__|	|__|__|__|__| |__|__|__|__| |__|__|__|__|	|__|__| |__|__| |__|__|	|__|__| |__|__| |__|__|

Have you received an x-ray examination of the…	Yes	No	Don't know	How often in total	In which year, beginning with the most re-cent one?	At what age?	How often?
**Thoracic region** (Lungs, Heart)	☐1	☐2	☐-8	|__|__|	|__|__|__|__| |__|__|__|__| |__|__|__|__|	|__|__| |__|__| |__|__|	|__|__| |__|__| |__|__|
**Abdominal region** (Kidneys, ureter, urinary bladder, gastrointestinal tract)	☐1	☐2	☐-8	|__|__|	|__|__|__|__| |__|__|__|__| |__|__|__|__|	|__|__| |__|__| |__|__|	|__|__| |__|__| |__|__|
Ribs	☐1	☐2	☐-8	|__|__|	|__|__|__|__| |__|__|__|__| |__|__|__|__|	|__|__| |__|__| |__|__|	|__|__| |__|__| |__|__|
Spine, whole	☐1	☐2	☐-8	|__|__|	|__|__|__|__| |__|__|__|__| |__|__|__|__|	|__|__| |__|__| |__|__|	|__|__| |__|__| |__|__|
Cervical spine	☐1	☐2	☐-8	|__|__|	|__|__|__|__| |__|__|__|__| |__|__|__|__|	|__|__| |__|__| |__|__|	|__|__| |__|__| |__|__|
Thoracic spine	☐1	☐2	☐-8	|__|__|	|__|__|__|__| |__|__|__|__| |__|__|__|__|	|__|__| |__|__| |__|__|	|__|__| |__|__| |__|__|
Lumbar spine	☐1	☐2	☐-8	|__|__|	|__|__|__|__| |__|__|__|__| |__|__|__|__|	|__|__| |__|__| |__|__|	|__|__| |__|__| |__|__|
Shoulder/ Upper arm	☐1	☐2	☐-8	|__|__|	|__|__|__|__| |__|__|__|__| |__|__|__|__|	|__|__| |__|__| |__|__|	|__|__| |__|__| |__|__|
Elbow/forearm/<br>Wrist	☐1	☐2	☐-8	|__|__|	|__|__|__|__| |__|__|__|__| |__|__|__|__|	|__|__| |__|__| |__|__|	|__|__| |__|__| |__|__|

<table>
<tr><th>Did you have an x-ray examination of the…</th><th>Yes</th><th>No</th><th>Don’t know</th><th>How often in total</th><th>In what year, beginning with the most re-cent one?</th><th>At what age?</th><th>How often?</th></tr>
<tr><td>Hand/finger</td><td>☐1</td><td>☐2</td><td>☐-8</td><td>|__|__|</td><td>|__|__|__|__|
__	__	__	__
__	__	__	__	</td><td>	__	__
__	__
__	__	</td><td>	__	__
__	__
__	__	</td></tr>
<tr><td>Pelvis/Hip</td><td>☐1</td><td>☐2</td><td>☐-8</td><td>	__	__	</td><td>	__	__	__	__
__	__	__	__
__	__	__	__	</td><td>	__	__
__	__
__	__	</td><td>	__	__
__	__
__	__	</td></tr>
<tr><td>Femur/Knee</td><td>☐1</td><td>☐2</td><td>☐-8</td><td>	__	__	</td><td>	__	__	__	__
__	__	__	__
__	__	__	__	</td><td>	__	__
__	__
__	__	</td><td>	__	__
__	__
__	__	</td></tr>
<tr><td>Lower leg/Ankle</td><td>☐1</td><td>☐2</td><td>☐-8</td><td>	__	__	</td><td>	__	__	__	__
__	__	__	__
__	__	__	__	</td><td>	__	__
__	__
__	__	</td><td>	__	__
__	__
__	__	</td></tr>
<tr><td>Foot/Toes</td><td>☐1</td><td>☐2</td><td>☐-8</td><td>	__	__	</td><td>	__	__	__	__
__	__	__	__
__	__	__	__	</td><td>	__	__
__	__
__	__	</td><td>	__	__
__	__
__	__	</td></tr>
<tr><td>Other x-ray examinations: ____________________</td><td>☐1</td><td>☐2</td><td>☐-8</td><td>	__	__	</td><td>	__	__	__	__
__	__	__	__
__	__	__	__	</td><td>	__	__
__	__
__	__	</td><td>	__	__
__	__
__	__	</td></tr>
<tr><td>Bone density measurements, this is an examination to determine whether one has osteoporosis (bone loss).</td><td>☐1</td><td>☐2</td><td>☐-8</td><td>	__	__	</td><td>	__	__	__	__
__	__	__	__
__	__	__	__	</td><td>	__	__
__	__
__	__	</td><td>	__	__
__	__
__	__	</td></tr>
<tr><td>For women only: Mammography, this is an examination of the breasts.</td><td>☐1</td><td>☐2</td><td>☐-8</td><td>	__	__	</td><td>	__	__	__	__
__	__	__	__
__	__	__	__	</td><td>	__	__
__	__
__	__	</td><td>	__	__
__	__
__	__	</td></tr>
</table>

Further x-ray examinations:

Body part	In which year?	At what age?	How often?

Space for interviewer's comments:

X-ray examinations

B2.0 Did you ever have an x-ray examination during which a contrast agent was introduced?

Contrast agents can be drunk or swallowed, injected via a blood vessel or introduced as an enema.

They aid the visualization of hollow organs such as the gastrointestinal tract, the bile duct or blood vessels, allowing them to be examined better.

☐1 **Yes** ⇨ continue with B2.1

☐2 **No** ⇨ continue with C1.0

☐-8 **Don't know** ⇨ continue with C1.0

B2.1 What kinds of examinations were conducted?

I read to you different examination possibilities. Please answer to each item.

Info for interviewer: Please go through the list of body parts, even if the subject names a specific examination right away.

For each examination the total number will be asked first, followed by the respective year, beginning with the most recent (e.g. 2013).

**If the subject cannot remember the year use the probing question: How old were you (in years)?*

For paper version: Where there are more than 3 examination years per examination, please enter the other years in the area "further examinations" at the end of the section.

Did you have an x-ray examination with a contrast agent of the…	Yes	No	Don't know	How often in total	In which year, beginning with the most recent one?	At what age, respectively?	How often?
Esophagus	☐1	☐2	☐-8	|__|__|	|__|__|__|__| |__|__|__|__| |__|__|__|__|	|__|__| |__|__| |__|__|	|__|__| |__|__| |__|__|
Stomach/Small intestine	☐1	☐2	☐-8	|__|__|	|__|__|__|__| |__|__|__|__| |__|__|__|__|	|__|__| |__|__| |__|__|	|__|__| |__|__| |__|__|
Colon	☐1	☐2	☐-8	|__|__|	|__|__|__|__| |__|__|__|__| |__|__|__|__|	|__|__| |__|__| |__|__|	|__|__| |__|__| |__|__|

Gallbladder and biliary tract (ERCP)	☐1	☐2	☐-8	|__|__|	|__|__|__|__
__	__	__	__
__	__	__	__			__	__
__	__
__	__			__	__
__	__
__	__	
**Did you have an x-ray examination with a contrast agent of the…**	**Yes**	**No**	**Don't know**	**How often in total**	**In which year, beginning with the most recent one?**	**At what age?**	**How often?**
Kidney and urinary tract	☐1	☐2	☐-8	|__|__|	|__|__|__|__
__	__	__	__
__	__	__	__			__	__
__	__
__	__			__	__
__	__
__	__	
Blood vessels (Veins, arteries)	☐1	☐2	☐-8	|__|__|	|__|__|__|__
__	__	__	__
__	__	__	__			__	__
__	__
__	__			__	__
__	__
__	__	
Spinal canal	☐1	☐2	☐-8	|__|__|	|__|__|__|__
__	__	__	__
__	__	__	__			__	__
__	__
__	__			__	__
__	__
__	__	
Joints	☐1	☐2	☐-8	|__|__|	|__|__|__|__
__	__	__	__
__	__	__	__			__	__
__	__
__	__			__	__
__	__
__	__	
Uterus, ovaries	☐1	☐2	☐-8	|__|__|	|__|__|__|__
__	__	__	__
__	__	__	__			__	__
__	__
__	__			__	__
__	__
__	__	
**Other:**<br>**What was examined?**<br>(e.g. upper gastro-intestinal tract)<br>______________________	☐1	☐2	☐-8	|__|__|	|__|__|__|__
__	__	__	__
__	__	__	__			__	__
__	__
__	__			__	__
__	__
__	__	

Further x-ray examinations using a contrast agent:

Body part	In which year?	At what age?	How often?

Space for interviewer's comments:

Interventional radiology

C1.0 Did you ever have cardiac catherization?

During cardiac catheterization the doctor inserts a thin tube (catheter) through a blood vessel in the groin (or less commonly in the elbow) into the body. Inside the body, the catheter is pushed through the blood vessels to the heart. The doctor monitors the procedure constantly on the x-ray screen. She/he can examine the pumping function of the heart or dilate narrowed blood vessels of the heart by inflating a small balloon or inserting so-called stents. Cardiac catheterization is generally performed following a heart attack or prior to heart surgeries.

☐1 **Yes** ⇨ continue with *C1.1*

☐2 **No** ⇨ continue with *C2.0*

☐-8 **Don't know** ⇨ continue with *C2.0*

Info for interviewer: Please go through the list of body parts, even if the subject names a specific examination right away.

For each examination the total number will be asked first, followed by the respective year, beginning with the most recent (e.g. 2013).

**If the subject cannot remember the year use the probing question: How old were you (in years)?*

For paper version: Where there are more than 3 examination years per examination, please enter the other years in the area "further examinations" at the end of the section.

C1.1

How often in total	In which year, beginning with the most recent one?	At what age?	How often?
|__|__|	|__|__|__|__|	|__|__|	|__|__|
	|__|__|__|__|	|__|__|	|__|__|
	|__|__|__|__|	|__|__|	|__|__|

Further cardiac catherization:

In which year?	At what age?	How often?

Space for interviewer's comments:

C2.0 **Did you ever have other so-called minimal-invasive radiological procedures?**

Here I mean procedures which are conducted under radiologic control, for example the insertion of stents into a blood vessel, in the respiratory tract or the bile duct to keep them "open". Or did you ever have an embolization done?

☐1 **Yes** ⇨ continue with C2.1

☐2 **No** ⇨ continue with D0

☐-8 **Don't know** ⇨ continue with D0

Info for interviewer: Please go through the list of body parts, even if the subject names a specific examination right away.

For each examination the total number will be asked first, followed by the respective year, beginning with the most recent (e.g. 2013).

**If the subject cannot remember the year use the probing question: How old were you (in years)?*

For paper version: Where there are more than 3 examination years per examination, please enter the other years in the area "further examinations" at the end of the section.

C2.1

Which procedure?	Body part	How often in total?	In which year?	At what age?	How often?
		|__|__|	|__|__|__|__| |__|__|__|__| |__|__|__|__|	|__|__| |__|__| |__|__|	|__|__| |__|__| |__|__|
		|__|__|	|__|__|__|__| |__|__|__|__| |__|__|__|__|	|__|__| |__|__| |__|__|	|__|__| |__|__| |__|__|
		|__|__|	|__|__|__|__| |__|__|__|__| |__|__|__|__|	|__|__| |__|__| |__|__|	|__|__| |__|__| |__|__|
		|__|__|	|__|__|__|__| |__|__|__|__| |__|__|__|__|	|__|__| |__|__| |__|__|	|__|__| |__|__| |__|__|
		|__|__|	|__|__|__|__| |__|__|__|__| |__|__|__|__|	|__|__| |__|__| |__|__|	|__|__| |__|__| |__|__|

Further minimal-invasive procedures:

Procedure	Body part	In which year?	At what age?	How often?

Space for interviewer's comments:

Nuclear medicine examinations

D0 **I now come to nuclear medicine examinations. These examinations show organ dysfunctions e.g. of the thyroid, lungs, bones or the heart.**

In most cases the patient is injected with a low-radioactive agent. The agent reaches the organ of interest through the blood vessels. A computer generates an image which is generally multicolored, a so-called scintigram. The medical terms for these nuclear medicine examinations are: scintigraphy, PET or SPECT.

PET (Positron-Emission-Tomography)

SPECT (Single photon emission computed tomography)

➔*If necessary, show image*

Did you ever have a nuclear medicine examination?

☐1 **Yes** ⇨ continue with D1.0

☐2 **No** ⇨ continue with E0

☐-8 **Don't know** ⇨ continue with E0

D1.0 Which body organ/part was examined by scintigraphy, PET or SPECT?

Info for interviewer: Please go through the list of body parts, even if the subject names a specific examination right away.

For each examination the total number will be asked first, followed by the respective year, beginning with the most recent (e.g. 2013).

**If the subject cannot remember the year use the probing question: How old were you (in years)?*

For paper version: Where there are more than 3 examination years per examination, please enter the other years in the area "further examinations" at the end of the section.

	Yes	No	Don't know	How often in total	In which year, beginning with the most recent one?	At what age?	How often?
**Lungs**	☐1	☐2	☐-8	|__|__|	|__|__|__|__| |__|__|__|__| |__|__|__|__|	|__|__| |__|__| |__|__|	|__|__| |__|__| |__|__|
**Heart**	☐1	☐2	☐-8	|__|__|	|__|__|__|__| |__|__|__|__| |__|__|__|__|	|__|__| |__|__| |__|__|	|__|__| |__|__| |__|__|
**Kidneys**	☐1	☐2	☐-8	|__|__|	|__|__|__|__| |__|__|__|__| |__|__|__|__|	|__|__| |__|__| |__|__|	|__|__| |__|__| |__|__|
**Thyroid gland**	☐1	☐2	☐-8	|__|__|	|__|__|__|__| |__|__|__|__| |__|__|__|__|	|__|__| |__|__| |__|__|	|__|__| |__|__| |__|__|
**Bones**	☐1	☐2	☐-8	|__|__|	|__|__|__|__| |__|__|__|__| |__|__|__|__|	|__|__| |__|__| |__|__|	|__|__| |__|__| |__|__|
**Other body parts?** ________________	☐1	☐2	☐-8	|__|__|	|__|__|__|__| |__|__|__|__| |__|__|__|__|	|__|__| |__|__| |__|__|	|__|__| |__|__| |__|__|

Further nuclear medicine examinations:

Body part	In which year?	At what age?	How often?

Space for interviewer's comments:

Nuclear medicine examinations

D1.1 *If „nuclear medicine examinations (Question D0)“ ever answered yes:*

Do you consent that we contact your radiologist or treating physician to ask for additional information regarding the type of examination?

Yes ☐1 **No** ☐2

Please specify the name and address of the practice or clinic in which the nuclear medicine examination was conducted.

Name of practice or clinic: ____

Street: ____

Zip code/ City: ____

**In case the name of the practice or clinic is not recalled, please ask for the name of the referring physician (e.g. general practitioner).*

Name of referring physician: ____

Street: ____

Zip code/ City: ____

Therapies

E0 We now come to the last section of this questionnaire. It is on radiation therapies which are conducted for example in cases of thyroid diseases or cancer.

Did you ever have thyroid radioiodine therapy?

For thyroid radioiodine therapy the patient has to be admitted into hospital . A radioactive agent (iodine) is injected. The patient has to be isolated from other people until the level of the agent in the body has gone down. This therapy is only performed in specialized hospital departments or radiology departments.

- ☐ 1 **Yes** ⇨ continue with E1.0
- ☐ 2 **No** ⇨ continue with F0
- ☐ -8 **Don’t know** ⇨ continue with F0

Info for interviewer: Please go through the list of body parts, even if the subject names a specific examination right away.

For each examination the total number will be asked first, followed by the respective year, beginning with the most recent (e.g. 2013).

**If the subject cannot remember the year use the probing question: How old were you (in years)?*

For paper version: Where there are more than 3 examination years per examination, please enter the other years in the area “further examinations” at the end of the section.

E1.0

How often in total?	In which year, beginning with the most recent one?	At what age?	How often?
|__|__|	|__|__|__|__| |__|__|__|__| |__|__|__|__|	|__|__| |__|__| |__|__|	|__|__| |__|__| |__|__|

Further thyroid radioiodine therapy examinations:

In which year?	At what age?	How often?

Space for interviewer’s comments:

Therapies

F0	**Did you ever have radiotherapy?**
	During radiotherapy radiation is used for treatment, for example of cancer. In a few cases benign diseases such as arthritis, calcaneal spurs or Bekhterev's disease are also treated using radiotherapy.
	☐ 1 **yes** ⇨ continue with F1.0
	☐ 2 **no** ⇨ End of interview
	☐ -8 **don't know** ⇨ End of interview

F1.0

	**1**. Examination	**2.** Examination	**3.** Examination
**Reason for radiotherapy (e.g. kind of tumor)**	________________	________________	________________
**When was this?** (Year of therapy start)	|__|__|__|__| Year	|__|__|__|__| Year	|__|__|__|__| Year
Alternatively: How old were you at therapy start?	|__|__| Age	|__|__| Age	|__|__| Age

Further radiation therapy:

**Examination (Reason for radiotherapy)**	**Year of therapy start**	**At what age?**	**How often?**

Space for interviewer's comments:

F2.0 *If radiotherapy (question F0) ever answered yes:*

Do you consent that we contact your radiologist or treating physician to ask for additional information regarding the type of therapy and radiation dose?

Yes ☐ 1 **No** ☐ 2

Please specify the name and address of the practice or clinic in which the nuclear medicine examination was conducted.

Name of practice or clinic: ____

Street: ____

Zip code/ City: ____

**In case the name of the practice or clinic is not recalled, please ask for the name of the referring physician (e.g. general practitioner).*

Name of referring physician: ____

Street: ____

Zip code/ City: ____

Thank you for taking the time to participate in the interview!

End of interview „Medical Exposure“: ____**:**____

As you have read in the study information, for the following 6 months we would like to ask you to hand over an x-ray card to the attending physician at each x-ray examination, which was specially developed for the German National Cohort. Your physician is asked to provide some features of the examinations, which will be used to derive radiation doses from. After the 6-month period, we kindly ask you to send the x-ray cards back to us in the envelope we have provided.

**X-ray card accepted:**	**Yes**	☐ 1	**No**	☐ 2

Special incidents:

____

____

____

____
